# Bacterial community adaptation after brackish and freshwater coalescence

**DOI:** 10.1093/ismeco/ycag198

**Published:** 2026-07-10

**Authors:** Xiu Jia, Torsten Schubert, Rick Beeloo, Aristeidis Litos, Swapnil Doijad, Pim G van Helvoort, Theodor Sperlea, Matthias Labrenz, Bas E Dutilh

**Affiliations:** Institute of Biodiversity, Ecology, and Evolution, Faculty of Biological Sciences, Cluster of Excellence Balance of the Microverse, Friedrich Schiller University Jena, Jena, 07745, Germany; Institute of Biodiversity, Ecology, and Evolution, Faculty of Biological Sciences, Cluster of Excellence Balance of the Microverse, Friedrich Schiller University Jena, Jena, 07745, Germany; Department of Biology, Science4Life, Utrecht University, Padualaan 8, Utrecht, CH 3584, the Netherlands; Institute of Biodiversity, Ecology, and Evolution, Faculty of Biological Sciences, Cluster of Excellence Balance of the Microverse, Friedrich Schiller University Jena, Jena, 07745, Germany; Institute of Biodiversity, Ecology, and Evolution, Faculty of Biological Sciences, Cluster of Excellence Balance of the Microverse, Friedrich Schiller University Jena, Jena, 07745, Germany; Department of Biology, Science4Life, Utrecht University, Padualaan 8, Utrecht, CH 3584, the Netherlands; Department of Biological Oceanography, Leibniz Institute for Baltic Sea Research Warnemünde, Seestraße 1, Rostock, 18119, Germany; Department of Biological Oceanography, Leibniz Institute for Baltic Sea Research Warnemünde, Seestraße 1, Rostock, 18119, Germany; Institute of Biodiversity, Ecology, and Evolution, Faculty of Biological Sciences, Cluster of Excellence Balance of the Microverse, Friedrich Schiller University Jena, Jena, 07745, Germany; Department of Biology, Science4Life, Utrecht University, Padualaan 8, Utrecht, CH 3584, the Netherlands

**Keywords:** environmental selection, time series, community dynamics, Oxford Nanopore Technologies, estuary

## Abstract

Microbial community coalescence, the merging of entire microbial communities, is common in estuaries, yet the ecological processes governing community assembly after coalescence remain unclear. To uncover these mechanisms, we conducted controlled microcosm experiments simulating coalescence between freshwater and brackish water bacterial communities across five mixing ratios and incubated them in either freshwater or brackish water. We tracked 40 mixed communities over six passages using Nanopore full-length 16S rRNA gene sequencing. Laboratory incubation reduced diversity and increased taxonomic overlap, but preserved habitat-associated compositional signatures. Coalescence outcomes were asymmetric and strongly shaped by the incubation water. In both environments, coalesced communities remained compositionally closer to the native than to the introduced community. However, the relative influence of environmental filtering differed between habitats. In freshwater, strong filtering drove communities to closely resemble the freshwater source, even when the freshwater inoculum was small. In brackish water, weaker filtering made coalescence outcomes more dependent on initial mixing ratios and propagule pressure. Neutral model analyses confirmed a greater contribution of stochastic processes in brackish water. Co-occurrence network analysis revealed predominantly positive associations within the same source community and fewer but negative associations across sources. These within-source associations may reinforce propagule pressure by facilitating the establishment of co-adapted species. Overall, our results demonstrate that environmental filtering primarily governs coalescence outcomes, modulated by source composition, stochasticity, and species co-adaptation, offering new insights into microbial assembly in dynamic estuarine systems.

## Introduction

Dispersal contributes to homogenizing microbial communities across habitats [1]. Due to their microscopic size, microbes often disperse not as individual cells but as an entire community. When previously separated microbial communities come into contact and mix, a process known as community coalescence occurs [2, 3]. Community coalescence has been observed in diverse systems, including flooding events, soil tillage, estuarine mixing, wastewater treatment, and microbiome transplantation, and is now increasingly recognized as a relevant concept in microbial ecology.

Once coalescence takes place, the newly formed community undergoes rapid restructuring, often leading to the emergence of an alternative community. The outcomes of this process can vary: in some cases, both source communities contribute equally, resulting in a symmetric structure; whereas in others, one dominates, leading to an asymmetric structure [4]. These outcomes are shaped by multiple factors, including filtering by the new physicochemical conditions, the mixing ratio of source communities, the strength/volume of community exchange, and the temporal dynamics of coalescence events [2, 5]. Moreover, post-coalescence dynamics may also be influenced by priority effects [6] and even newly formed species interactions [7], all of which modulate how communities reassemble and stabilize after merging.

Changes in salinity, pH, and nutrient availability can strongly select microbial taxa. For example, bacterial communities are highly sensitive to salinity disturbances in aquatic environments [8, 9]. Driven by differences in growth rate and genomic adaptability, species have different niche breadths and may respond differently to environmental changes. Specialist taxa such as *Roseovarius* are uniquely adapted to high-salinity environments [10], while generalists with a broad niche breadth can persist across a wide range of habitats [11]. For example, marine generalists can persist in freshwater and retrieve their abundance when exposed to brackish water [12]. Yet, the extent to which environmental filtering influences species survival and community structure after coalescence is still insufficiently understood.

In addition to environmental filtering, the size of the source communities may influence coalescence outcomes. Larger single-species populations are more likely to establish successfully due to reduced effects of demographic stochasticity, causing propagule pressure [13, 14]. Likewise, a larger, diverse community also has a higher chance of establishing [15]. However, communities are not just the sum of independent populations. Processes such as cross-feeding or mutual facilitation among taxa can facilitate reestablishment during community assembly. Co-selection has been observed between dominant and rare species through cross-feeding during coalescence [16]. Moreover, larger community sizes are associated with a greater number of taxa [15], potentially enhancing opportunities for interspecies interactions. Nonetheless, empirical evidence for the influence of community size on coalescence outcomes remains limited.

Estuarine ecosystems provide an ideal habitat to study microbial community coalescence. This habitat experiences continuous mixing of freshwater and seawater communities along salinity gradients. Riverine inputs transport eutrophic, freshwater-associated microbiomes into the coastal sea, while tidal intrusions carry oligotrophic, marine-associated microbiomes upstream. These microbiomes are typically compositionally and phylogenetically distinct [17], and their mixing creates transition zones where both environmental conditions and microbial communities shift [18, 19]. Salinity, in particular, has been identified as a major environmental driver influencing estuarine microbial communities, along with dissolved organic matter (DOM), temperature, and pH [20–22]. For example, *Synechococcus* tends to increase with rising salinity [23]. Studies in the Baltic Sea have also shown that marine species increase in abundance with rising salinity, while freshwater species decline. For example, populations of *Candidatus* Pelagibacter ubique (SAR11) and *Synechococcus* exhibited phylogenetic shifts along the salinity gradient [19]. Although these studies demonstrate that salinity influences community composition, the mechanisms driving community shifts during coalescence in estuaries are still not well understood. This is especially pressing as estuarine ecosystems are particularly sensitive to sea-level rise [24].

A small but growing number of studies have begun to reveal how diversity, stability, and dominance patterns emerge after microbial community coalescence. These include work on soil bacterial communities [25–27], bacterial communities in aquaculture systems [28], and estuarine microeukaryotic communities [29]. However, experimental studies that directly follow bacterial temporal responses to coalescence, and the factors that shape their assembly in estuarine systems, remain limited. To address this gap, we experimentally mixed natural freshwater and brackish water bacterial communities in defined proportions under controlled conditions. Using microcosm experiments, we tracked community dynamics over time to address two key questions: How do environmental conditions and source community mixing ratios influence community establishment following coalescence? Which species survive or thrive in the newly coalesced communities, and do some respond consistently depending on their source?

## Materials and methods

### Field sample collection

We selected bacterial communities from the estuarine environment because estuaries provide a freshwater to brackish water transition gradient, and their conditions can be easily replicated in the laboratory using a liquid medium. On 20 April 2023, we collected surface water samples from the Warnow Estuary (freshwater) and the adjacent Baltic Sea coast (brackish water), where salinity ranged from 0.41 to 13.96 parts per thousand. These two sampling sites correspond to site 1 and site 9 of a project investigating the spatiotemporal dynamics of microbial communities along a ~30 km transect across the Warnow estuary and the adjacent Baltic Sea coast [30]. At each site, we collected 5 L of surface water (~0–30 cm depth) and kept the samples at 4°C before transporting them to the laboratory for further processing. We recorded sampling time, geographic coordinates, and *in situ* measurements of conductivity (an indicator of salinity), temperature, and pH using a conductivity-temperature-depth sensor (Supplementary Table S1). These environmental measurements are part of the published dataset [30].

### Coalescence experiment setup in microcosms

To investigate the influence of coalescence ratios and environmental conditions on bacterial community dynamics after coalescence, we established microcosms with five different mixing ratios of freshwater and brackish water bacterial communities under two environmental conditions (filtered freshwater and brackish water).

**Figure 1:**
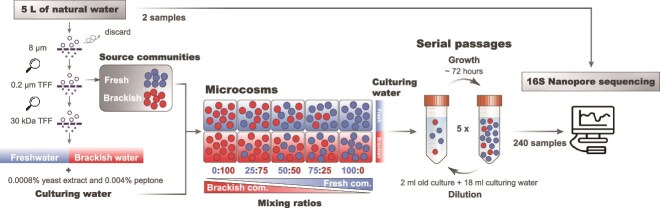
Schematic overview of the experimental setup. Microcosms were inoculated with an original or mixed community in an organism-free freshwater or brackish water medium. We mixed bacterial communities from freshwater (blue dots) and brackish water (red dots) sources to obtain five mixing ratios. These mixed communities were cultured in either freshwater (blue background) or brackish water (red background). Different combinations of the mixed microbial community and the environment are shown in squares (microcosms). Communities were transferred to fresh medium at each passage; this process was repeated five times, resulting in six passages. Changes in community composition were tracked using full-length 16S rRNA gene sequencing on a Nanopore platform.

The initial freshwater and brackish water bacterial communities were prepared by stepwise filtration (Fig. 1, left panel). First, organisms larger than 8 μm were removed from 5 L of water using a bottle-top filtration system. This pre-filtration step excludes most protistan grazers (e.g. heterotrophic nanoflagellates), enriching for the free-living bacterial fraction and minimizing top-down control during subsequent incubations [31]. The bacterial fractions were then concentrated 25-fold to a final volume of 200 ml using a 0.2 μm modular tangential flow filtration (TFF) system (Vivaflow®, Sartorius, Göttingen, Germany). The filtrate from the 0.2 μm TFF was further processed through a 30 kDa modular TFF (Vivaflow®, Sartorius, Göttingen, Germany) to remove living organisms, including ultra-small bacteria, phages, and vesicles. The resulting filtered freshwater and brackish water were used as the culturing water in the microcosms. The filtrate was not autoclaved to preserve the *in situ* characteristics of the growth medium, as heat- and pressure-induced hydrolysis of DOM and abiotic release of labile substrates can artificially stimulate bacterial growth and bias experimental outcomes [32]. To ensure sufficient microbial biomass for analysis, we supplemented the filtered freshwater and brackish water with a limited amount of sterile yeast extract (Carl ROTH®, Karlsruhe, Germany) and peptone (Carl ROTH®, Karlsruhe, Germany), with final concentrations of 0.0008% yeast extract and 0.004% peptone.

Community coalescence was established by mixing freshwater and brackish water bacterial communities at five volumetric ratios (0:100, 25:75, 50:50, 75:25, 100:0 $v/v$; Fig. 1, center panel) on 24 April 2023. To initiate community coalescence, each microcosm was prepared by adding 2 ml of either the mixed or unmixed source community to 18 ml of filtered water (freshwater or brackish water), for a total volume of 20 ml. Each treatment was independently replicated four times, yielding a total of 40 microcosms. Here, 100% freshwater communities in filtered freshwater and 100% brackish water communities in filtered brackish water were referred to as reference controls for the source communities.

Microcosms were incubated in dark conditions at 20°C in an Eppendorf Innova 44® incubation shaker, with continuous shaking at 200 rpm under standard atmospheric pressure and oxygen levels. The incubation temperature was chosen to simulate peak *in situ* summer conditions at the sampling site [30], thereby optimizing biomass recovery. Bacterial communities in our microcosms reached a relatively stable cell density within ~72 h, as determined by OD600 and SYBR green measurements (Supplementary Fig. S1). Accordingly, 72 h was used as the standard interval between passages, except for passage 1, which was sampled at 92 h after inoculation. We note that this interval reflects stabilization of overall biomass rather than confirmed compositional stability, as community composition was not assessed over the 72 h. At each transfer, 2 ml (10% of the culture volume) was inoculated into 18 ml of filtered freshwater or brackish water medium to initiate the next passage. This transfer process was repeated five times, resulting in a total of six passages. At each passage, an uninoculated freshwater/brackish water medium control (*n* = 1) was incubated under the same conditions as a medium negative control (Supplementary Fig. S2). Although the medium was filter-sterilized, a small fraction of ultra-small or filter-passing bacteria can remain. For example, *Hylemonella gracilis*, a bacterium with cell sizes <0.1 μm, can pass our 0.2 μm filters [33]. Likewise, lineages such as *beta proteobacterium* CB, reported in freshwater systems as members of low-nucleic-acid, streamlined or very small-cell-size lineages [34], may also pass through 0.2 μm filters. These bacteria were detected only at extremely low abundance in our microcosm samples and did not influence the interpretation of our results.

At the end of each passage, before the next transfer, 10 ml of each microcosm was collected to monitor community dynamics over time. Samples were centrifuged at 13 000 × g for 2 min to pellet bacterial cells, and the supernatant was discarded. To maintain the integrity of the total microbial community and minimize loss of loosely associated biomass during additional handling, pellets were not washed but were instead immediately air-dried in a laminar flow hood and stored at −80°C for subsequent DNA extraction.

### DNA extraction and full-length 16S PCR sequencing

Bacterial pellets were resuspended in TE buffer, and cells were lysed stepwise using lysozyme, proteinase K, and the lysis solution CBO from the smart DNA prep (m) DNA extraction kit (IST Innuscreen GmbH, Berlin, Germany). DNA extraction was performed according to the manufacturer’s instructions using an automated liquid handling platform, CyBio FeliX (Analytik Jena GmbH, Jena, Germany). Following chemical lysis, AMPure XP beads (Beckman Coulter Inc., USA) were added to purify DNA. DNA from the two source communities was extracted by first pre-filtrating 2 L of freshwater and brackish water through 8 μm membranes to remove large particles and organisms, followed by collection of bacterial fraction onto 0.2 μm membranes using a bottle-top filtration system, and subsequent extraction using the DNeasy PowerWater Kit (QIAGEN, Germany). DNA concentration was quantified for each sample using the DeNovix dsDNA High Sensitivity Kit (DeNovix, Wilmington, USA) and CLARIOstar^Plus^ plate reader (BMG Labtech, Ortenberg, Germany).

We used full-length 16S rRNA gene sequencing to profile bacterial community composition, as it contains more information for highly resolved phylogenetic classification. Recent studies have demonstrated that Nanopore R10.4.1 provides a rapid, efficient, and affordable method for microbial identification in complex environmental samples [35]. The bacterial full-length 16S rRNA gene was amplified using the forward primer 27F (5′-AGRGTTYGATYMTGGCTCAG-3′) and reverse primer 1492R (5′-CGGYTACCTTGTTACGACTT-3′). Both primers were tagged with 17 barcodes modified from the barcode list from the Barcoding Expansion 1–96 kit (ONT, EXP-PBC096, Oxford Nanopore Technologies, Oxford, UK). A combination of these barcoded primers enables us to process up to 289 samples (see sample list with barcodes in Supplementary Data S1).

For each 16S amplification reaction, we combined 2 μl of sample DNA (average concentration ± standard error: 2.13 ± 0.16 ng/μl) with 1.25 μl of each primer (10 μM), 0.5 μl dNTPs (10 mM), and 0.25 μl Q5 High-Fidelity DNA Polymerase (New England Biolabs) for a final volume of 25 μl. To avoid single-amplification bias, each sample was amplified in three technical replicates, and the replicates were pooled before further purification and sequencing. PCR amplification was performed using a Biometra TAdvanced thermal cycler (Analytik Jena, Jena, Germany), with the following cycling conditions: 5 min at 98°C to activate polymerase; 10 s at 98°C for denaturation, 30 s at 60°C for annealing, 2 min at 72°C for extension (5 cycles); 10 s at 98°C for denaturation, 2.5 min at 72°C for extension (30 cycles); and a final step of 2 min at 72°C. Potential contamination was checked by including a no-template control on each PCR plate and verifying it with gel electrophoresis. Negative PCR controls were also sequenced and yielded only a negligible number of reads compared to the biological samples (Supplementary Fig. S2).

Following PCR, amplicons were purified first using AMPure beads to remove primers, dNTPs, polymerase, and other PCR reagents. Samples were quantified using the DeNovix dsDNA High Sensitivity Kit and pooled to achieve the same molecular weight. To select our target amplicons (~1500 bp) and remove non-target fragments, the pooled sample was further purified using the Zymoclean Gel DNA Recovery Kit (ZYMO RESEARCH, Freiburg, Germany).

The final pooled 16S rRNA library, including all study samples and ZymoBIOMICS Microbial Community Standards (even and log distributions; ZYMO Research, Freiburg, Germany), was sequenced in a single run using the Ligation Sequencing Kit V14 (SQK-LSK114, Oxford Nanopore Technologies, Oxford, UK) on one FLO-MIN114 flow cell (R10.4.1 nanopore). The ZymoBIOMICS Microbial Communities were included as independent samples to assess the performance of PCR amplification and nanopore sequencing [36]. All expected bacterial genera from the two ZymoBIOMICS microbial communities were successfully recovered, and the observed community composition closely reflected the expected distribution, although some deviations were present (Supplementary Fig. S3). This indicates that our sequencing run can reliably recover the bacterial community structure, although with some variance.

### Sequence processing

After sequencing, we obtained 14.29 M raw pod5 reads (24.03 Gbases) from 272 samples, comprising 240 microcosm samples, 18 negative controls, 6 mock communities, and 8 field samples. We used Dorado (version 0.9.1) to convert raw Nanopore signals to FASTQ files. After basecalling, 14 565 367 fastq reads were retained for further analysis with a median read length of 1554 nt and a median read quality score of 13.9. Demultiplexing was performed using Barbell [37], which uses Sassy to detect adapter and primer sequences flanking each read [38]. The raw sequence with the adapter and primer was then compared against all barcode sequences, and a barcode assignment was made when a match was detected within an edit distance of ≤5. Since the expected 16S amplicon length was 1300–1500 nt and dual-end barcoding was used, reads were assigned only when two barcode matches were identified within this expected distance of one another. To account for concatemer formation, reads containing multiples of this pattern were split accordingly. Overall, 6 391 864 reads were successfully demultiplexed into 272 samples. The demultiplexed fastq reads were taxonomically annotated using Emu, which uses the expectation–maximization algorithm to generate species-level annotations [39]. We retained a total of 5 474 871 bacterial reads from 1065 species across 242 samples (240 microcosm samples and two natural samples), with per-sample read counts ranging from 3712 (minimum) to 83 381 (maximum), a median of 20 202, and a mean of 22 623.

### Data transformation and diversity analysis

All further data analyses were performed using R (version 4.3.3). The rarefaction curves of microcosm samples reached a plateau, indicating that their diversity was sufficiently captured (Supplementary Fig. S4).

To assess how laboratory conditions influenced source community composition, we compared the log_2_ fold-change in relative abundance between natural and laboratory conditions in both freshwater and brackish water habitats. To identify freshwater and brackish water bacterial species, we performed differential abundance analysis of these species between freshwater and brackish water communities under laboratory conditions using the ANOVA-Like Differential Expression tool (ALDEx2), which is based on center-log ratio (CLR) transformed data. Species richness was analyzed by fitting a Generalized Linear Mixed Model (GLMM) with a Poisson distribution and log-link function using the lme4 package. Mixing ratio, culturing water, passage number, and all their associated interactions were included as fixed effects. To control for temporal repeated measures, the replicate was defined as a random intercept. Fixed-effects significance was determined via Type III Analysis of Deviance Wald chi-square tests using the car package, while *post hoc* linear trends and passage-specific pairwise contrasts with Tukey adjustments were computed using the emmeans package. To address the compositional nature of amplicon sequencing data, we applied a CLR transformation to nonzero read counts by the geometric mean across all species in a sample. Community dissimilarity was then quantified by calculating the Aitchison distance, the Euclidean distance between the CLR-transformed abundance of species across samples.

To identify co-occurrence relationships among bacterial species during community coalescence, we constructed a co-occurrence network using SpiecEasi v1.0.7 [40] with the Meinshausen–Bühlmann (MB) neighborhood selection method. Species present in fewer than four samples were excluded before analysis, retaining 401 species across 240 samples. Count data were CLR-transformed before network inference as described above. Network sparsity was determined using the bounded Stability Approach to Regularization Selection (bSTARS) criterion [41] with 20 subsampling replicates, a stability threshold of 0.05, and 120 lambda values (lambda.min.ratio = 0.01); the achieved stability score was 0.048, indicating robust edge selection. Edge weights correspond to the symmetrized partial correlation coefficient (|β|) between pairs of species estimated by the MB model. To assess whether the observed network topology differed from random expectations, we generated 999 null networks using degree-preserving edge rewiring [42], performing 10 times the number of edges as rewiring attempts per null graph, and compared observed modularity, clustering coefficient, mean path length, and betweenness centrality against the null distribution using z-scores. Modules within the network were identified using the Louvain community detection algorithm. The network was visualized using a Fruchterman–Reingold layout, with node fill color indicating source community enrichment as determined by ALDEx2 (see above), node border color indicating module membership, and node size reflecting mean CLR-transformed relative abundance across all samples. The proportions of positive and negative interactions within and between modules and source community enrichment groups were summarized to assess whether the co-occurrence structure reflected source community identity.

## Results and discussion

This study provides an empirical analysis of coalescence between freshwater and brackish water bacterial communities under controlled experimental conditions. Using a Nanopore-based full-length 16S rRNA analysis pipeline, we resolved community dynamics at species-level resolution, enabling mechanistic insights into the influence of environmental filtering, stochasticity, and species interactions during coalescence of complex aquatic microbial communities.

### Distinct taxonomic composition of source communities

**Figure 2:**
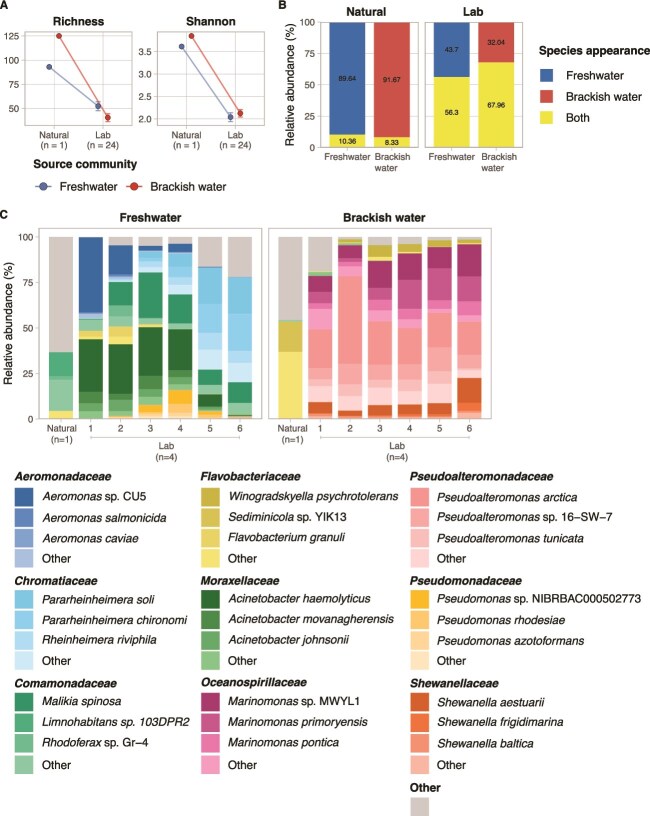
Species-level composition of bacterial communities in freshwater and brackish water under natural and laboratory conditions. (A) Richness and Shannon diversity of two different source communities under two growth conditions. For microcosm samples, error bars represent the standard error of the mean based on three biological replicates through six sampling time points (*n* = 24). Field samples represent single *in situ* measurements (*n* = 1). (B) Total relative abundance of bacterial species detected in both freshwater and brackish water, or exclusive to one habitat under natural or laboratory conditions. (C) Stacked bar plots showing the relative abundance of species (the top three species within each of the nine most abundant families) in freshwater and brackish water under natural (*n* = 1) and laboratory conditions (mean of four replicates). The x-axis shows the natural samples followed by the sequential passages of lab samples.

The two source communities showed compositional divergence in their natural environments, with limited taxonomic overlap. In samples collected from upstream of the Mühlendamm on the Warnow River (freshwater) and the adjacent Baltic Sea coast at Strand Warnemünde (brackish water), we detected a total of 200 bacterial species, of which only 18 were shared (Fig. 2A and Supplementary Fig. S5B). Habitat-unique species contributed around 90% of the total read abundance in each habitat, while shared species represented ~8%–10% of the sequencing reads (Fig. 2B). The natural freshwater community was dominated by taxa such as *Limnohabitans* and *Polynucleobacter* (Supplementary Data S2, Fig. 2C), consistent with their widespread prevalence across diverse freshwater habitats [43, 44]. The brackish water community was dominated by taxa such as *Planktomarina, Formosa*, and *Candidatus* Pelagibacter ubique, which are characteristic of brackish and marine environments and adapted to oligotrophic conditions [19, 45–47]. These compositional differences corroborate previous work showing that microbial community structure shifts along estuaries, where changes are often related to salinity transitions [48, 49].

### Laboratory conditions reduce diversity but preserve habitat signatures of source communities

**Figure 3:**
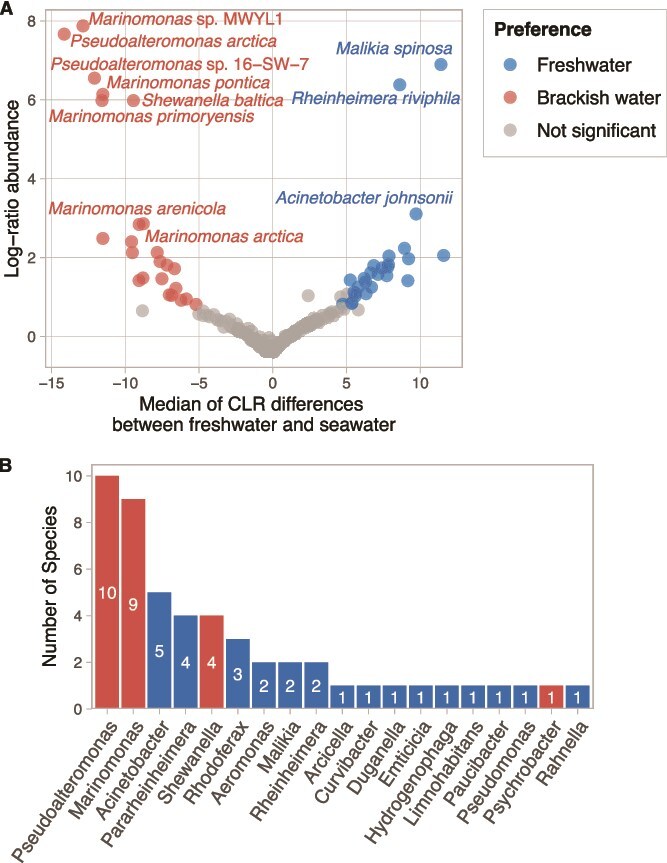
Freshwater and brackish water preferred species under laboratory conditions. (A) Differential abundance of species between freshwater and brackish water communities under laboratory conditions, based on ALDEx2 analysis incorporating all passages and replicates (*n* = 24). The x-axis represents the effect size, defined as the median difference in CLR-transformed abundance between the two source communities, and the y-axis shows the median CLR-transformed abundance across all samples. Colored dots indicate species with significant differences (adjusted *P*-value <.05), based on the Wilcoxon rank-sum test and Benjamini–Hochberg correction. Species are labeled for those with log-ratio abundance >2.5. (B) The number of differentially abundant species across enriched genera in the two environments. The bars and integer labels represent the total number of species belonging to that genus that were significantly enriched in the respective environment. Genera are ordered by their total species count. The complete ALDEx2 results, supporting both panels (A) and (B), are provided in Supplementary Data S3.

To investigate the ecological mechanisms underlying coalescence, we conducted experiments under controlled laboratory conditions, minimizing the environmental complexity inherent in field environments. Source communities were subjected to the same environmental treatments, enabling direct comparison of mixed and unmixed communities under a common baseline. Both freshwater and brackish water communities showed reduced diversity compared to their natural counterparts (Fig. 2A). In addition to this cultivation-associated loss of diversity, species richness declined significantly across passages following coalescence in both culturing waters (Supplementary Fig. S5A; GLMM passage effect: *χ*^2^ = 5.37, *P* = .021; Supplementary Tables S2 and S3), although the rate and direction of change depended on the initial inoculum composition and culturing water. While local sample richness declined, the absolute regional pool of species detected across all combined laboratory samples was numerically higher than in the natural samples. This is an artifact of expended sampling effort (*n* = 24; Supplementary Fig. S5B), rather than an expansion of community richness. Most species present in natural environments were depleted under laboratory conditions, and only a few became enriched (Supplementary Fig. S6A). Laboratory incubation also increased taxonomic overlap between freshwater and brackish water communities compared to natural samples, with 33 species shared, accounting for 56%–67% of the total sequence abundance (Fig. 2B, Supplementary Fig. S5B). Despite these changes, habitat-associated compositional signatures were preserved, as confirmed by ALDEx2 differential abundance analysis. In laboratory freshwater, 27 species were significantly enriched, including *Malikia spinosa* and *Rheinheimera* spp. (Figs 2C, 3A, Supplementary Figs S7–S8, Supplementary Data S3), both of which are characteristic of freshwater habitats [50, 51]. In laboratory brackish water, 25 species were enriched, including *Marinomonas* spp., *Pseudoalteromonas* spp., and *Shewanella baltica*, taxa with well-documented associations with brackish and marine environments [52, 53]. These species-level enrichments are consistent with genus-level patterns, where *Malikia, Pararheinheimera, Acinetobacter*, and *Rheinheime*ra were more abundant in freshwater, while *Pseudoalteromonas, Marinomonas*, and *Shewanella* were more abundant in brackish water (Supplementary Fig. S9, Supplementary Data S4).

Collectively, these results indicate that while laboratory cultivation conditions reduced overall diversity and increased taxonomic overlap between source communities, they retained habitat-adapted taxa and preserved the distinctive ecological signature of source communities. Several experimental factors likely contributed to the observed diversity loss and taxonomic overlap. Filtration-induced removal of larger particles, particle-associated microorganisms [54], and DOM likely contributed directly to diversity loss, as DOM is a key substrate structuring microbial community composition [55]. Although dissolved organic carbon was measured only in field samples and not in microcosm samples (Supplementary Table S1), filtration likely partially homogenized substrate availability between freshwater and brackish water media, potentially contributing to the increased taxonomic overlap observed between the two communities under laboratory conditions. Despite experimental modification, habitat-specific bacterial taxa were retained in both freshwater- and brackish water-derived media. While filtration might remove particulate material, dissolved-phase characteristics of the source waters, such as ionic composition and DOM, should be largely preserved. These retained physicochemical characteristics likely supported the persistence of habitat-associated taxa despite the reduction in overall diversity. In addition, shaking-induced homogenization of microscale gradients and the addition of low levels of labile nutrients (0.0008% yeast extract and 0.004% peptone) likely promoted opportunistic taxa, consistent with the observed enrichment of taxa with faster growth rates (Supplementary Fig. S6B). These trade-offs are inherent to microcosm experiments, which sacrifice environmental complexity in favor of mechanistic clarity under controlled and replicable conditions [56]. Thus, our findings should be interpreted as mechanistic insights into the ecological processes governing microbial community dynamics after coalescence, rather than as direct predictions of compositional outcomes in natural estuaries. Nevertheless, the persistence of habitat-associated taxa and consistent enrichment patterns suggests that the underlying mechanisms remain relevant beyond the controlled setting.

### Environmental conditions strongly influence community coalescence outcomes

**Figure 4:**
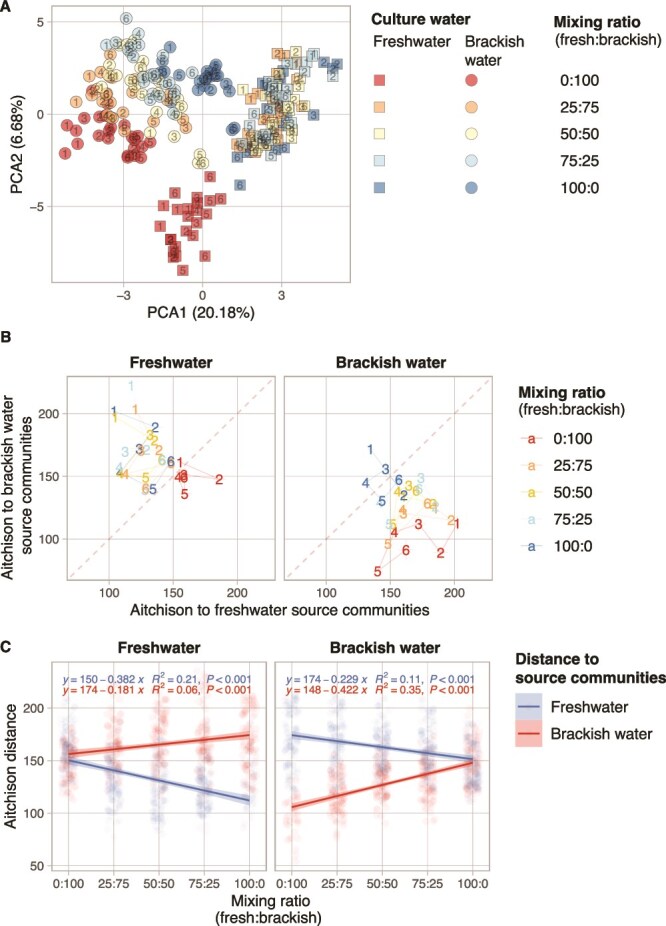
Community dissimilarity after mixing based on Aitchison distance at the species level. (A) Compositional principal component analysis (PCA) biplot based on Euclidean distance of the CLR-transformed feature table for PCA. Axes show the percentage of total variance explained by the first two components. Colors represent the mixing ratios of freshwater and brackish water communities; shapes represent the growth environment; numbers inside the shapes represent passages. (B) Dissimilarity (Aitchison distance) between mixed communities and their freshwater or brackish water source communities. Colors represent mixing ratios; numbers represent passages. Points/Numbers represent the mean dissimilarity of the mixed community to the two source communities; error bars represent the standard error. Lines connect temporal changes across passages for each group. (C) Regression of Aitchison distance between mixed communities and their source communities as a function of the proportion of the source communities in the initial mix. Freshwater source communities are 100% freshwater communities cultured in freshwater; brackish water source communities are 100% brackish water communities cultured in brackish water. The y-axis shows the Aitchison distance of the coalesced communities to the source communities. The x-axis indicates the initial mixing ratios of freshwater and brackish water communities before culturing, with four replicates per ratio across six passages. Colors represent the source community. The left and right panels correspond to the culturing environments of mixed communities (freshwater or brackish water). Regression equations, *R*^2^ values, and *P*-values are displayed for each group in the corresponding panel.

The structure of the coalesced bacterial communities was strongly influenced by the aquatic growth environments, as evidenced by the clear separation of samples from different culturing waters in a principal component plot (Fig. 4A). PERMANOVA confirmed that environmental conditions were the dominant driver of community variation (*R*^2^ = 14.55, *P* < .001), followed by mixing ratio (*R*^2^ = 10.07, *P* < .001), and passages (*R*^2^ = 4.91, *P* < .001; Supplementary Table S4). Significant but weak interaction effects were also observed between mixing ratio and environment (*R*^2^ = 6.78, *P* < .001) and between passage and environment (*R*^2^ = 4.00, *P* < .001), indicating that the influence of inoculum composition and temporal changes varied across environmental context. Because communities were repeatedly transferred into the same culturing medium at each passage, abiotic conditions remained relatively constant throughout the experiment, ensuring that the observed environmental effect reflects sustained, consistent selective pressure rather than a transient response.

In the freshwater environment, communities containing 25%–100% freshwater bacteria clustered together. Only communities containing 0% freshwater bacteria (i.e. 100% brackish water bacteria) formed a distinct group. These results suggest that the freshwater medium imposed a strong environmental filtering for pre-adapted taxa, such that even a small freshwater inoculum was sufficient to drive coalesced communities toward the freshwater compositional state. This is supported by species richness dynamics in freshwater, where the pure brackish water inoculum initially had significantly lower richness than all freshwater-containing mixing ratios at passage 1 (Tukey-adjusted *P*  $\le$ .016, Supplementary Table S5, Supplementary Fig. S5A), but recovered over passages, converging with all other mixing ratios by passage 6 (all *P* > .05). This recovery pattern further suggests that the water conditions select for freshwater-adapted taxa regardless of starting composition. The recovery may also have been facilitated by freshwater bacteria already present in the brackish inoculum due to riverine inputs at the Baltic Sea coast [57]. In addition, the freshwater source contained approximately three-fold higher bacterial cell density than the brackish source [30], increasing the possibility of the freshwater community establishment even when introduced at low mixing proportions.

In the brackish water environment, we observed a gradual shift in community structure across mixing ratios (Fig. 4A), indicating both inoculum composition and weaker environmental filtering jointly shaped the final community structure. This lack of recovery may also be due to a three-fold lower bacterial cell density observed in the brackish water source [30], resulting in a sparse seed bank that impeded reestablishment of brackish-adapted species. Species richness mirrored this pattern, i.e. the pure freshwater inoculum had significantly lower richness than all other mixing ratios at passage 1 (Tukey-adjusted *P*  $\le$ .015, Supplementary Table S5, Supplementary Fig. S5A), but by passage 6, the pure brackish water inoculum became significantly lower than all other mixing ratios (all *P*  $\le$ .005), possibly due to a dilution-to-extinction effect resulting from the lower cell density of the brackish source community. However, the coalesced community in brackish water did not converge toward the freshwater composition despite the higher cell density of the freshwater community inoculum, indicating that cell density alone cannot explain the outcome and that weak selection by environmental conditions in brackish water still plays a role.

The stronger filtering in freshwater may reflect tighter physiological constraints imposed by low-salinity conditions, which select for species of narrowly adapted freshwater specialists. In contrast, the brackish zone is characterized by frequent mixing and dispersal from both freshwater and marine sources, favoring generalist and broadly adapted species with wider niche breadths and thereby weakening the apparent strength of filtering [58, 59]. Together, these patterns suggest that an interplay between the strength of environmental filtering and inoculum proportion governs coalescence outcomes. In freshwater, strong filtering and high cell density enable even a relatively small inoculum to reestablish the original community. The situation differs in brackish water, where weaker filtering and/or low cell density make the final community composition sensitive to the initial mixing ratio.

Our findings provide further evidence for the importance of environmental filtering in governing microbial community composition during coalescence. Similar patterns have been reported in aquatic systems, where mixing freshwater and marine water resulted in brackish conditions that act as a strong environmental filter, causing extensive loss of taxa and driving community convergence toward marine-adapted microbiomes [60]. Likewise, in soil systems, where coalescence typically involves the simultaneous mixing of microbial communities and their resident environments in equal proportions, a recent study has also identified abiotic factors as the main drivers of community variation [61]. This happened when one soil imposed abiotic dominance, likely due to its higher clay and organic matter contents, a higher pH, and greater cation exchange capacity, which buffered its physicochemical conditions during coalescence and disproportionately favored the establishment of pre-adapted taxa [61]. In our experiment, environmental filtering was likely underestimated compared with oligotrophic natural estuarine systems. The addition of low concentrations of yeast extract and peptone to filtered freshwater and brackish water partially reduced nutrient differences between habitats, while filtration may have further homogenized substrate availability by removing particles and associated DOM. Although these experimental manipulations likely changed several abiotic factors, the limited water volume in the microcosm experiment precluded measurements of abiotic variables such as salinity, pH, DOM, and inorganic nutrients across passages. Consequently, we could not identify which specific factors primarily drove environmental filtering. Still, the remaining differences in background water abiotic conditions were sufficient to preserve the habitat-specific signatures of source communities and contributed to the stronger signal of selection observed in freshwater. Broader comparison thus supports the hypothesis that when one microbial community is introduced into the environmental setting of another, environmental filtering favors already-adapted resident bacteria, thereby promoting asymmetric coalescence outcomes in which residents outperform invaders [4].

### Source community contributions vary with environmental conditions

To quantify the relative contribution of each source community to coalescence outcomes, we calculated Aitchison distances between coalesced communities and each source community. In both cultivation environments, coalesced communities were consistently more similar to the native source community than to the introduced one (Fig. 4B). This further supports the concept of an asymmetric coalescence outcome, in which the establishment of species from another biome is limited [4]. A temporal trend was observed only in freshwater, where the distance between coalesced and brackish water source communities gradually decreased over passages, whereas the distance to the freshwater source remained largely unchanged, suggesting progressive displacement of introduced brackish water species under sustained freshwater selection (Fig. 4B). When fitted against the mixing ratio, the distance to the native source community decreased as its inoculum proportion increased, whereas the distance to the introduced source community showed the opposite pattern, increasing slightly with its inoculum proportion (Fig. 4C). These patterns further explained the observed asymmetry in community coalescence, namely, environments preferentially select for native, well-adapted species, and increasing the initial abundance of the native community further reinforces its dominance in the coalesced community. This dependence on mixing ratio was especially pronounced in brackish water, where the regression fit was stronger (*R*^2^ = 0.35, *P* < .001) than with freshwater (*R*^2^ = 0.21, *P* < .001), consistent with the principle that weak environmental filtering allows initial source community proportions to influence more on coalescence outcomes, whereas strong environmental filtering drives communities toward a single environmentally determined state regardless of starting proportions [61, 62].

The influence of the initial proportion of each source community on coalescence outcomes is related to propagule pressure, as higher propagule pressure reduces the risk of stochastic extinction and increases the probability that introduced species reach densities sufficient to compete for available niches [63]. Recent experimental and modeling work also demonstrates that coalescence outcomes can follow a dose-dependent response, in which interspecific competition for shared resources reduces resource availability and amplifies the advantage of the initially dominant community [64]. Notably, the weaker competitor shows a stronger dependence on its initial dose, an effect that becomes more pronounced as community diversity increases [64]. In short, these findings highlight that coalescence outcomes are shaped not only by environmental filtering but also by the initial abundance of source communities, with propagule pressure exerting greater influence when environmental filtering is relaxed, as observed in brackish water.

### Stochastic processes also contribute to coalescence outcomes, particularly in brackish water

**Figure 5:**
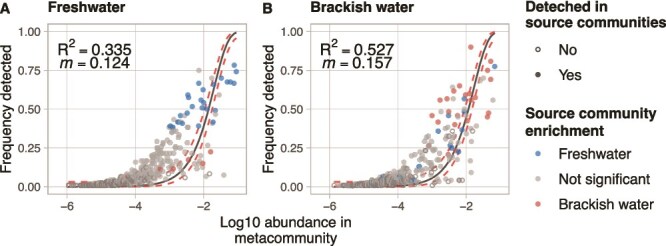
Neutral processes influence the structure of communities. Sloan neutral model prediction of meta-communities growing in freshwater (A) and brackish water (B), respectively. Values of *R*^2^ indicate the fit of the model, and *m* represents the estimated dispersal rate among communities. The dark grey solid line represents the model prediction, and the red dashed lines represent the frequency of occurrence within the 95% confidence interval.

To assess the extent to which deviations from strong environmental filtering could be explained by stochastic processes, we applied the Sloan neutral models to bacterial communities that developed in two environments. Communities growing in brackish water had a higher neutral model fit (*R*^2^ = 0.527) than those growing in freshwater (*R*^2^ = 0.335; Fig. 5), suggesting that stochastic processes such as reproduction, deaths, and immigration play a greater role in shaping communities in brackish water than in freshwater. Consistently, the estimated dispersal rate was also higher in brackish water (*m* = 0.157) than in freshwater (*m* = 0.124), reinforcing the stronger influence of initial source community proportions on the final community structure in the brackish water relative to freshwater. Additionally, more species in freshwater fell above the neutral model prediction interval, indicating their occurrence frequencies were higher than expected given their abundances, consistent with stronger environmental selection in freshwater than in brackish water. This higher neutral model fit and dispersal rate in brackish water align with the weaker environmental filtering and provide a mechanistic basis for why coalesced community composition in brackish water remains sensitive to initial source community proportions rather than converging on a single environmentally determined state. Notably, the relative contribution of stochastic processes may be overestimated in our system, particularly in brackish water, where natural communities are more oligotrophic, as nutrient addition is known to decrease deterministic selection and increase the importance of stochastic processes [65, 66]. Stochastic processes are therefore likely weaker in the oligotrophic natural systems than in our experiment.

### Bacteria from their native environments maintain covariation during coalescence

**Figure 6:**
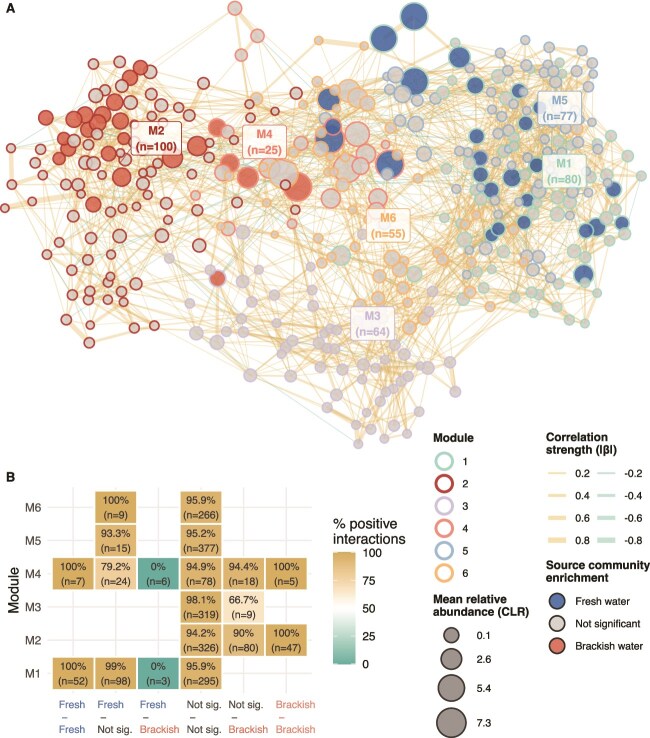
Co-occurrence network of bacterial species across all microcosm samples. (A) The network was generated using SpiecEasi (Meinshausen–Bühlmann method, bSTARS model selection; stability score = 0.048) and visualized with a Fruchterman–Reingold layout. Nodes represent bacterial species (*n* = 401); edges indicate significant partial correlations inferred by the network model (*n* = 2040). Node border color indicates module membership as determined by the Louvain community detection algorithm. Node size reflects mean relative abundance (CLR-transformed) across all samples. Node fill color indicates enrichment in source communities as determined by ALDEx2 differential abundance analysis: blue shows freshwater-enriched, red shows brackish water-enriched, and grey shows not significantly enriched. Edge color indicates the direction of the association: gold indicates positive co-occurrence; teal indicates negative co-occurrence. Edge width corresponds to the strength of the partial correlation (|β|). (B) Heatmap showing the percentage of positive interactions between species pairs grouped by module and source community enrichment pairing. Tiles represent the proportion of positive edges out of all edges between each enrichment pairing; total edge counts are shown in parentheses. Color scale ranges from teal (0% positive, all negative) through white (50%) to gold (100% positive, all positive).

To investigate whether bacterial species from distinct source communities maintain ecological associations during coalescence, we constructed a co-occurrence network using SpiecEasi (stability score = 0.048; Fig. 6) across all microcosm samples (401 species, 2040 edges). The resulting network showed strong deviations from random expectations, with significantly higher modularity (Q = 0.529, *z* = 44.96, *P* < .001), clustering coefficient (z = 69.04, *P* < .001), and higher betweenness centrality (z = 138.16, *P* < .001), indicating pronounced local clustering and a compartmentalized topology with highly connected species. Our network contained six modules. Freshwater- and brackish water-enriched species were predominantly assigned to distinct modules (Fig. 6A, Supplementary Fig. S10A), suggesting that species from the same habitat origin co-vary during coalescence. Consistent with this, edges connecting pairs of species that were enriched in the same habitat were exclusively positive, while the edges between freshwater- and brackish water-enriched species were all negative (Fig. 6B). This suggests shared environmental adaptation and/or facilitative interactions among species from the same source habitat, versus differential environmental responses and/or competitive exclusion among species from different habitats. These within-source positive associations may further reinforce the influence of initial source community proportions on coalescence outcomes by enabling groups of co-adapted species to establish collectively and amplify the effects of propagule pressure. It should be noted that co-occurrence networks reflect statistical associations rather than direct ecological interactions, and the role of rare species in mediating network structure and coalescence dynamics remains difficult to resolve with the current approach [67, 68].

The emergence of modular co-occurrence patterns, where habitat-matched species co-occur, is consistent with observations in mixed microbial communities, where functionally or ecologically compatible groups form tightly linked modules [69]. Such modularity likely originated from differences among source communities, rather than among the newly formed coalesced community [4]. The strong positive associations within source-community-enriched species may reflect both shared environmental adaptations and cooperative interactions [68, 70]. Within the brackish water module, *Marinomonas* and *Pseudoalteromonas* have consistently been found to co-occur as predominant taxa driving alginate degradation [71], suggesting functional complementarity as a potential mechanism underlying their positive association. Within the freshwater module, *Rhodoferax* and *Curvibacter* have been shown to utilize carbon fractions and metabolic byproducts released by *Limnohabitans* [72]. Although direct pairwise interaction data for the species in our network were not available in the BactInt or mi-atlas databases [73, 74], these examples provide ecological support for the within-habitat positive associations observed here. The negative associations between freshwater and brackish water modules are consistent with differential habitat filtering, as these taxa occupy fundamentally distinct osmotic niches and are unlikely to co-exist under either environmental condition. Competitive exclusion driven by niche overlap among ecologically similar but habitat-differentiated species may further contribute [75]. Our findings also align with theoretical predictions that cooperative interactions promote species establishment during coalescence, whereas competitive interactions constrain it [76]. Moreover, interactions among community members may become increasingly important under the stress imposed by coalescence, as stressful conditions can strengthen species dependencies and promote facilitative interactions [77]. Consistent with this idea, bacterial invaders often establish more successfully when introduced alongside compatible taxa that facilitate colonization through cross-feeding or environmental modification [78, 79]. Collectively, these results suggest that coalescence outcomes could be shaped by both environmental filtering acting on individual species and the network of ecological interactions within the source communities.

### Extrapolating from microcosm to field: opportunities and constraints

Our microcosm experiment was designed to isolate and identify the ecological mechanisms governing community coalescence under controlled conditions. First, we must caution that coalescence outcomes in natural estuaries will not necessarily mirror those observed here, even if the underlying mechanisms may be transferable. In natural estuaries, strong salinity and nutrient gradients interact with continuous mixing, variable residence times, and spatially patchy hydrodynamics, making communities that may shift rapidly and in complex ways. Unlike our constant-temperature microcosms, freshwater and brackish water masses in natural systems vary in temperature with daily and seasonal cycles, which may influence coalescence outcomes by affecting growth rates, species interactions, and the strength of environmental filtering [80]. Furthermore, natural coalescence involves the simultaneous mixing of microbial communities and their environmental matrix, including nutrients, salinity, and pH. Future field-based studies should account for this mixing of environmental factors, as the context arriving with an introduced community may itself modify local selective pressures. Priority effects represent a further complexity absent from our design. Because all communities were introduced simultaneously, our experimental setup minimized priority effects by design. In nature, however, arrival history can matter. Taxa introduced earlier may pre-empt resources or modify local conditions before complete mixing occurs, particularly where residence times are long enough to permit growth. Conversely, turbulence and tidal exchange can repeatedly disrupt these legacies by accelerating coalescence and resetting local composition [81]. Our results also highlight priorities for future coalescence studies. Disentangling the contributions of environmental filtering and species interaction would benefit from unamended controls in which source communities are mixed without nutrient addition, isolating the effect of source community identity from copiotroph enrichment. Nutrient enrichment is known to intensify competitive interactions [82], which may inflate interaction strength inferred from co-occurrence networks. Omitted here to enable the growth of sufficient biomass for DNA extraction and sequencing, nutrient-free cultivation would be more realistic, even though all laboratory experiments necessarily remain divergent from the natural system. Finally, our study focused exclusively on bacterial communities and did not capture interactions with other organisms, such as fungi, protists, or bacteriophages, all of which can substantially alter bacterial community dynamics, e.g. through predation and lysis. Future studies integrating multi-trophic interactions, environmentally realistic abiotic gradients, and long-term field observations will be essential to refine the general ecological rules governing microbial coalescence and its implications for estuarine ecosystem functioning.

## Conclusions

This study provides mechanistic insights into bacterial community coalescence across a freshwater–brackish water interface. Using species-level resolution under controlled experimental conditions, we demonstrated that coalescence outcomes are strongly asymmetric, consistently favoring native over introduced taxa. Strong environmental filtering in freshwater drove communities to converge toward a freshwater-like community even with a small amount of freshwater inoculum, whereas weaker filtering in brackish water allowed stochastic dynamics and propagule pressure to exert greater influence on final community structure. Network analysis further revealed that source community identity was preserved during coalescence, with positive associations among taxa from the same habitat and negative associations across habitats. These network findings likely reinforce the effects of propagule pressure by enabling groups of co-adapted species to establish collectively. While our microcosm experiments necessarily simplify the environmental complexity of natural estuaries, the ecological mechanisms identified here are likely relevant beyond the controlled setting. However, our design omitted variables such as environmental measurements, continuous mixing, temperature fluctuations, and multitrophic interactions; future studies integrating environmentally realistic abiotic gradients, unamended conditions, and field-based observations will be essential for assessing how these mechanisms scale to natural coalescence.

## Supplementary Material

Supplementary_material_ycag198

## Data Availability

Raw sequencing data generated and analyzed by this study are available at the European Nucleotide Archive (ENA) with the study accession number PRJEB103999. All other data and code are available at https://github.com/Jia-Xiu/coalescence_project.
