# Supplementary material for: Bacterial community adaptation after brackish and freshwater coalescence
Source: ISME Commun. 2026 Jul 10;6(1):ycag198. doi: 10.1093/ismeco/ycag198 (PMC13452456; doi:10.1093/ismeco/ycag198)
Supplement: Supplementary file 1 [file supplementary_material_ycag198.zip › Coalescence_supplementary_materials_Jia_et_al_02-07-2026.docx]

# Supplementary materials

For the manuscript:

**Bacterial community adaptation after freshwater and brackish water coalescence**

Xiu Jia, Torsten Schubert, Rick Beeloo, Aristeidis Litos, Swapnil Doijad, Pim G. van Helvoor, Theodor Sperlea, Matthias Labrenz, and Bas E. Dutilh

## Supplementary Figures

### **Figure S1. Cell density dynamics during passage 1**. To track the cell density of the community over time, we measured the optical density (OD600) and the fluorescent signal from SYBR Green dye in all microcosms at passage 1. Colors represent the mixing ratios of freshwater and brackish water source communities, culturing water types, and ddH2O blanks used for density measurements. Shapes represent experimental replicates. The left and middle columns correspond to different culturing water media.

### **Figure S2. Total raw read counts of each species in microcosm samples and negative controls.** (A) The rank-abundance curve of bacterial species across all microcosm samples (n = 240) is shown as dark gray points. Species detected in negative controls are shown in different colors: DNA extraction controls (green, n = 3), culturing medium controls (purple, n = 12; freshwater and brackish water media sampled across six passages), and PCR controls (orange, n = 3). Annotated bars indicate species that exceeded 5,000 reads in controls. (B) Zoomed-in view of panel (A). PCR amplification yielded a negligible number of reads compared to the biological sample. DNA extraction controls showed only trace contamination, at abundances much lower than in microcosm samples. Most species detected in the culturing medium controls had substantially fewer reads than in microcosm samples, except for a few ultra-small or filter-passing taxa (e.g. *Hylemonella gracilis*, *Methyloversatilis* sp. RAC08, and beta proteobacterium CB), indicating that although most microbes had been removed during medium preparation, some residual cells remained.

### **Figure S3. Positive controls of Nanopore sequencing.** Comparison of the relative abundance between the expected distribution of the ZymoBIOMICS™ microbial community standard and measured Nanopore sequencing results of the standard at the species level. (A) Stacked-bar plots and (B) scatter plots show the microbial community standard with even and log-abundance distributions. Each real sequencing result is represented by three replicates (r1-r3). Dashed lines in panel B indicate the perfect match between the measured and the expected distribution. Pearson’s correlation coefficient R and p values are labeled on the corresponding panel.

### **Figure S4. Rarefaction curves of microcosm and natural samples.** The x-axis shows sampling depth per sample, and the y-axis shows the corresponding number of detected species in all the annotated reads. Facet rows represent the initial inoculum proportions of freshwater and brackish water bacterial communities, while columns indicate the cultural medium of the mixed communities (freshwater or brackish water). Colors represent passages, with passage 0 corresponding to the pre-mixing natural samples.

### **Figure S5. Diversity of bacterial communities.** (A) Species richness across serial passages for each mixing ratio and culturing water after coalescence. Points show raw replicate values (n = 4); lines and shading represent 95% confidence intervals of estimated marginal means derived from a generalized linear mixed model with a Poisson distribution. The natural water sample (n = 1) is shown as Passage 0 but was not included in the models. (B) Venn diagram showing the overlap of 538 species detected across two different water sources and two growth conditions. In natural environments, 93 species were detected in freshwater and 125 in brackish water. Under laboratory conditions, 261 species were detected in freshwater and 154 in brackish water.

### **Figure S6. Natural- and lab-species in freshwater and brackish water source communities.** (A) Correlation of relative abundance (RA) between natural and laboratory conditions. Bacterial species were classified as natural- or lab if their log_2_ fold change between these two conditions is greater than 1 or less than -1; species without 2-fold changes were classified as non-preferential. Species with zero abundance were assigned a small pseudo-count (10^-6^) to enable the log_2_ transformation. Colors indicate preference groups, and open circles indicate species with RA < 0.1% in both conditions. (B) Predicted growth rates of bacterial species grouped by their preference, based on log_2_ fold changes. Numbers indicate how many species have been found in the codon use bias database for predicting growth rates [1].

### **Figure S7. Stacked barplot showing the relative abundances of the top nine bacterial families and the top four genera within each family across all communities.** Bar heights represent the relative abundance of each genus in individual samples. The x-axis shows the passage number of each replicate. Rows correspond to the cultural medium, in which the mixed communities were grown (freshwater or brackish water), and columns indicate the initial inoculum proportions of the freshwater and brackish water source communities.

### **Figure S8. Volcano plot showing the differential abundance of species between freshwater and brackish water communities across passages under laboratory conditions.** Results were based on ALDEx2 analysis comparing all passages and replicates between the two communities under laboratory conditions. The x-axis shows the median difference in CLR-transformed abundance between freshwater and brackish water, and the y-axis represents the adjusted *p*-value from the Wilcoxon rank-sum test. Colored dots indicate species with significant differences in either freshwater (blue) or brackish water (red), as determined by the Wilcoxon rank-sum test (adjusted *p* < 0.05). Each panel corresponds to a different passage.

### **Figure S9. Volcano plot showing the differential abundance of genera between freshwater and brackish water communities under laboratory conditions.** Results were based on ALDEx2 analysis comparing all passages and replicates between the two communities under laboratory conditions. The x-axis shows the median difference in CLR-transformed abundance between freshwater and brackish water, and the y-axis represents the adjusted *p*-value from the Wilcoxon rank-sum test. Colored dots indicate species with significant differences in either freshwater (blue) or brackish water (red), as determined by the Wilcoxon rank-sum test (adjusted p < 0.05). The complete ALDEx2 results are provided in [Supplementary Data S2](https://drive.google.com/file/d/1gTcSpD32dMYtn53Ve4BedZolsoA2DsV_/view?usp=sharing).

### **Figure S10. Species from source communities and keystone identification in the co-occurrence network.** (A) Species significantly enriched in freshwater or seawater source communities (based on ALDEx2 analysis), grouped by network module and taxonomic family. Bar length shows mean CLR abundance, bar color shows family. (B) Keystone species identified by degree and normalized betweenness centrality. Dashed lines indicate thresholds at 1.5 standard deviations above the mean. Species are classified as network hubs (high degree and high betweenness), connectors (high betweenness and moderate degree), module hubs (high degree and moderate betweenness), or peripheral species (low degree and low betweenness) [2]. Point size shows mean CLR abundance; point color indicates species classification. Labels highlight network hubs and module hubs or connector species with significant enrichment in freshwater or seawater source communities; label color indicates source community origin.

## Supplementary Tables

### **Table S1. Location and physicochemical characteristics of the two distinct water types used in this study.**

| **Original water sample** | **Freshwater** | **Brackish water** |
| --- | --- | --- |
| Latitude | 54°5'2'' N | 54°10'55'' N |
| Longitude | 12°9'6'' E | 12°4'46'' E |
| pH | 8.22 | 7.85 |
| Temperature (°C) | 10.7 | 6.7 |
| Conductivity (mS/cm) | 0.60 | 15.23 |
| Salinity (Practical Salinity Unit) | 0.60 | 15.22 |
| Dissolved organic carbon (mg/L) * | 11.10 | 4.24 |

* Dissolved organic carbon was quantified using a TOC analyzer (Shimadzu TOC-L-CPN H54114900072) on bacterial cell-free filtrates.

### **Table S2. Generalized linear mixed-effects model (GLMM) evaluating the effects of mixing ratio, culturing water, passage, and their interactions on bacterial richness.** The model was fitted with a Poisson error distribution and log link function, with replicate included as a random intercept to account for the repeated-measures design. Statistical significance was evaluated using Type III Wald *χ*² tests. Significance codes: *, *p* < 0.05; , *p* < 0.01; ***, *p* < 0.001.

| **Effect** | ***χ*²** | **df** | ***p*** | **Significance** |
| --- | --- | --- | --- | --- |
| **Mixing ratio** | 29.878 | 4 | <0.001 | *** |
| **Culturing water** | 27.355 | 1 | <0.001 | *** |
| **Passage** | 5.367 | 1 | 0.0205 | * |
| **Mixing ratio x Culturing water** | 54.795 | 4 | <0.001 | *** |
| **Mixing ratio x Passage** | 39.808 | 4 | <0.001 | *** |
| **Culturing water x Passage** | 76.782 | 1 | <0.001 | *** |
| **Mixing ratio x Culturing water x Passage** | 107.312 | 4 | <0.001 | *** |

### **Table S3. Estimated richness slopes across passages for each mixing ratio and watertype.** Slopes represent the estimated linear change in log-scale species richness per passage, derived from the fitted GLMM (Table S2). Positive values indicate increasing richness over passages; negative values indicate decline. Standard errors (SE) and 95% confidence intervals (CI) represent Wald asymptotic estimates of slope precision; individual trends are considered statistically significant if their 95% CI does not overlap with zero.

| **Watertype** | **Mix ratio** | **Slope  (log scale)** | **SE** | **Lower 95% CI** | **Upper 95% CI** |
| --- | --- | --- | --- | --- | --- |
| **Freshwater** | 0:100 | 0.0445 | 0.0192 | 0.0068 | 0.0821 |
|  | 100:0 | -0.0565 | 0.0166 | -0.089 | -0.0241 |
|  | 25:75 | -0.0828 | 0.0174 | -0.1169 | -0.0486 |
|  | 50:50 | -0.0217 | 0.0165 | -0.0541 | 0.0107 |
|  | 75:25 | -0.1004 | 0.0167 | -0.1331 | -0.0676 |
| **Brackish water** | 0:100 | -0.1949 | 0.0194 | -0.233 | -0.1568 |
|  | 100:0 | 0.0495 | 0.0201 | 0.0101 | 0.089 |
|  | 25:75 | -0.0754 | 0.0173 | -0.1094 | -0.0415 |
|  | 50:50 | -0.0111 | 0.0175 | -0.0455 | 0.0233 |
|  | 75:25 | -0.0117 | 0.0171 | -0.0452 | 0.0218 |

### **Table S4. Permutational Multivariate Analysis of Variance (PERMANOVA) results based on Aitchison distance of all bacterial communities in the microcosm experiment.** Each treatment factor was identified as a fixed component to the overall model; *p*-values were obtained using 999 permutations; *R*^2^ gives the estimated sizes of components of variation.

| **Factor** | **df** | ***R*^2^** | **Pseudo F** | ***p* value** |
| --- | --- | --- | --- | --- |
| **Culturing water** | 1 | 14.55 | 53.19 | <0.001 |
| **Mixing ratio** | 4 | 10.07 | 9.20 | <0.001 |
| **Passage** | 5 | 4.91 | 3.59 | <0.001 |
| **Mixing ratio x Culturing water** | 4 | 7.41 | 6.78 | <0.001 |
| **Mixing ratio x Passage** | 20 | 4.90 | 0.89 | 0.923 |
| **Culturing water x Passage** | 5 | 4.00 | 2.92 | <0.001 |
| **Mixing ratio x Culturing water x Passage** | 20 | 4.92 | 0.90 | 0.924 |
| **Residual** | 180 | 49.24 |  |  |
| **Total** | 239 | 100 |  |  |

### **Table S5. Post-hoc pairwise contrasts of estimated marginal means (emmeans) for species richness between mixing ratios at passage 1 and passage 6.** Marginal means were derived from a generalized linear mixed-effects model (GLMM; Table S2). Contrasts are expressed as back-transformed response ratios; values < 1 indicate lower species richness in the numerator mixing ratio. p-values were adjusted for multiple comparisons using the Tukey method. Significance codes: *** *p* < 0.001, ** *p* < 0.01, * *p* < 0.05, . *p* < 0.1, ns *p* ≥ 0.1.

| **Passage** | **Contrast between mix ratios** | **Response ratio** | **SE** | **z-ratio** | **p-value (Tukey)** | **Significance** |
| --- | --- | --- | --- | --- | --- | --- |
| **In freshwater** | | | | | | |
| 1 | 0:100 / 100:0 | 0.571 | 0.083 | -3.846 | 0.0011 | ** |
| 1 | 0:100 / 25:75 | 0.59 | 0.086 | -3.61 | 0.0028 | ** |
| 1 | 0:100 / 50:50 | 0.634 | 0.093 | -3.118 | 0.0157 | * |
| 1 | 0:100 / 75:25 | 0.519 | 0.075 | -4.516 | 1e-04 | *** |
| 1 | 100:0 / 25:75 | 1.033 | 0.146 | 0.231 | 0.9994 | ns |
| 1 | 100:0 / 50:50 | 1.11 | 0.157 | 0.738 | 0.9477 | ns |
| 1 | 100:0 / 75:25 | 0.909 | 0.128 | -0.681 | 0.9606 | ns |
| 1 | 25:75 / 50:50 | 1.075 | 0.153 | 0.506 | 0.9868 | ns |
| 1 | 25:75 / 75:25 | 0.879 | 0.124 | -0.91 | 0.8931 | ns |
| 1 | 50:50 / 75:25 | 0.818 | 0.116 | -1.419 | 0.6156 | ns |
| 6 | 0:100 / 100:0 | 1.993 | 0.294 | 4.669 | 0 | *** |
| 6 | 0:100 / 25:75 | 1.078 | 0.153 | 0.531 | 0.9842 | ns |
| 6 | 0:100 / 50:50 | 1.335 | 0.192 | 2.013 | 0.2595 | ns |
| 6 | 0:100 / 75:25 | 1.252 | 0.179 | 1.572 | 0.5152 | ns |
| 6 | 100:0 / 25:75 | 0.541 | 0.08 | -4.162 | 3e-04 | *** |
| 6 | 100:0 / 50:50 | 0.67 | 0.1 | -2.691 | 0.0553 | . |
| 6 | 100:0 / 75:25 | 0.628 | 0.093 | -3.133 | 0.0149 | * |
| 6 | 25:75 / 50:50 | 1.238 | 0.178 | 1.489 | 0.5698 | ns |
| 6 | 25:75 / 75:25 | 1.161 | 0.166 | 1.045 | 0.8341 | ns |
| 6 | 50:50 / 75:25 | 0.938 | 0.135 | -0.445 | 0.9919 | ns |
| **In brackish water** | | | | | | |
| 1 | 0:100 / 100:0 | 0.946 | 0.138 | -0.383 | 0.9954 | ns |
| 1 | 0:100 / 25:75 | 1.114 | 0.164 | 0.733 | 0.9487 | ns |
| 1 | 0:100 / 50:50 | 0.882 | 0.128 | -0.864 | 0.9101 | ns |
| 1 | 0:100 / 75:25 | 1.07 | 0.157 | 0.46 | 0.9908 | ns |
| 1 | 100:0 / 25:75 | 1.178 | 0.172 | 1.124 | 0.7937 | ns |
| 1 | 100:0 / 50:50 | 0.933 | 0.134 | -0.484 | 0.9888 | ns |
| 1 | 100:0 / 75:25 | 1.131 | 0.164 | 0.85 | 0.9148 | ns |
| 1 | 25:75 / 50:50 | 0.792 | 0.115 | -1.607 | 0.4928 | ns |
| 1 | 25:75 / 75:25 | 0.96 | 0.141 | -0.276 | 0.9987 | ns |
| 1 | 50:50 / 75:25 | 1.213 | 0.175 | 1.334 | 0.6698 | ns |
| 6 | 0:100 / 100:0 | 0.587 | 0.09 | -3.486 | 0.0045 | ** |
| 6 | 0:100 / 25:75 | 0.593 | 0.09 | -3.441 | 0.0052 | ** |
| 6 | 0:100 / 50:50 | 0.533 | 0.08 | -4.171 | 3e-04 | *** |
| 6 | 0:100 / 75:25 | 0.501 | 0.075 | -4.591 | 0 | *** |
| 6 | 100:0 / 25:75 | 1.011 | 0.149 | 0.073 | 1 | ns |
| 6 | 100:0 / 50:50 | 0.907 | 0.134 | -0.662 | 0.9644 | ns |
| 6 | 100:0 / 75:25 | 0.853 | 0.125 | -1.081 | 0.8165 | ns |
| 6 | 25:75 / 50:50 | 0.897 | 0.131 | -0.741 | 0.9469 | ns |
| 6 | 25:75 / 75:25 | 0.844 | 0.123 | -1.163 | 0.7723 | ns |
| 6 | 50:50 / 75:25 | 0.941 | 0.136 | -0.421 | 0.9934 | ns |

## References

1.	Weissman JL, Hou S, Fuhrman JA. Estimating maximal microbial growth rates from cultures, metagenomes, and single cells via codon usage patterns. *Proceedings of the National Academy of Sciences* 2021;**118**:e2016810118. <https://doi.org/10.1073/pnas.2016810118>

2.	Olesen JM, Bascompte J, Dupont YL *et al.* The modularity of pollination networks. *Proceedings of the National Academy of Sciences* 2007;**104**:19891-96. <https://doi.org/10.1073/pnas.0706375104>
